# Potential of citric and yeast waste fermentation products supplemented with fiber-degrading enzymes as alternatives to soybean meal in ruminant nutrition

**DOI:** 10.1186/s12917-025-05260-3

**Published:** 2026-01-21

**Authors:** Molthida Rungchaicharoenphai, Suphakon Pramotchit, Kannika Saisombut, Anusorn Cherdthong, Sawitree Wogtangtintharn, Chanon Suntara

**Affiliations:** Tropical Feed Resource Research and Development Center (TROFREC), Department of Animal Science, Faculty of Agriculture, Khon Kaen University, Khon Kaen, 40002 Thailand

**Keywords:** Citric waste, Yeast waste, Agri-byproduct, Fiber-degrading enzyme, Pellet product

## Abstract

**Background:**

This study aimed to investigate the effects of fiber-degrading enzyme supplementation in combination with citric waste fermented yeast waste (CWYW) as a replacement for soybean meal on ruminal fermentation characteristics, gas production kinetics, and digestibility. The in vitro gas production technique was used to evaluate these effects. A 2 × 4 + 1 factorial arrangement in a completely randomized design (CRD) was employed, resulting in nine treatments comprising either CWYW in powder or pellet form with varying enzyme levels (0%, 0.2%, 0.4%, and 0.6%), alongside a control diet containing full soybean meal.

**Results:**

The control diet yielded the highest cumulative gas production (*P* < 0.01), whereas the CWYW-powder without enzyme addition showed the highest gas production rate constant (*P* < 0.01). The in vitro dry matter digestibility (IVDMD) was also highest in the control group (*P* < 0.01), while organic matter digestibility (OMD) did not differ significantly among treatments. Increasing levels of fiber-degrading enzymes were associated with a linear decrease in pH at 48 h (*P* < 0.05) and a corresponding increase in NH₃-N concentrations (*P* < 0.05). However, there were no statistically significant differences among CWYW treatments in most parameters.

**Conclusion:**

Although the control diet performed best in terms of gas production and digestibility, the combination of CWYW and 0.4% fiber-degrading enzyme supplementation produced fermentation characteristics and digestibility values comparable to those of the control. These findings suggest that CWYW, particularly when supplemented with 0.4% enzyme, holds potential as a sustainable alternative to soybean meal in ruminant feed formulations.

## Introduction

Global citric acid production is estimated at approximately 2 million tons per year, with an annual growth rate of 3% [1]. China is the world’s leading producer and exporter, accounting for 59% and 74% of the global market, respectively. The production process involves microbial fermentation using *Aspergillus niger*, with cassava and cassava pulp mixed with rice bran as the primary substrates [2].

However, the process generates a substantial amount of residual waste, up to 69% of the input material, while only 11.5% becomes citric acid. This waste is acidic (pH ~ 4) and contains high moisture (77.6%) [3], making conventional disposal methods such as burning or landfilling environmentally problematic. Nonetheless, these residues still contain nutrients; crude protein content ranges from 3 to 7%, and the material is rich in structural carbohydrates such as neutral detergent fiber (NDF), acid detergent fiber (ADF), and acid detergent lignin (ADL), with values of 86.13%, 68.17%, and 19.74%, respectively [4, 5], indicating potential use as ruminant feed.

The global demand for livestock products is steadily increasing in parallel with population growth. The world population is projected to reach 9.8 billion by 2050, necessitating a 50% to 70% increase in food production to meet nutritional requirements [6, 7]. This rising demand has driven a corresponding expansion in global feed production. Growth is fueled by population growth, urbanization, and rising incomes, especially in developing countries, where meat consumption has surged by over 100 million tons since the late 1990 s [8, 9]. However, high-quality protein meals such as soybean meal and fish meal are still largely imported, with feed costs accounting for 60–70% of total livestock production expenses [10]. In many developing countries, total feed demand has increased sharply in recent years and is projected to rise further as livestock production expands. Despite this, ruminant feed accounts for only a small fraction of total feed production, underscoring the need for alternative, locally available feed resources to reduce reliance on costly imports and support national feed self-sufficiency goals.

High levels of citric acid residue in concentrate diets, however, may reduce digestibility, thus necessitating improvement through fermentation or enzyme treatment. The probiotic yeast *Saccharomyces cerevisiae* is known to enhance ruminal microbial activity and improve nutrient utilization in ruminants [10]. Yeast waste generated from ethanol production, which contains 60–70% yeast cells and 26–30% crude protein along with vitamins and minerals, has demonstrated potential as a protein-rich feed ingredient [11].

Citric waste fermented with yeast waste has been shown to replace up to 75% of soybean meal in concentrate diets without negative impacts on gas kinetics or in vitro digestibility [12]. Furthermore, supplementation with fiber-degrading enzymes improves fiber utilization by breaking down fiber bonds into fermentable monosaccharides, which are converted by ruminal microbes into volatile fatty acids (VFAs), the primary energy source for ruminants [13]. Although enzyme-treated citric waste has been reported to improve neutral detergent fiber (NDF) and acid detergent fiber (ADF) digestibility in beef cattle diets [14], it also reduced feed intake. Pelletizing the feed can address this issue by enhancing density, shelf life, and flowability, while reducing feed loss and enabling the use of diverse raw materials.

The utilization of citric acid residues as alternative feed ingredients not only addresses environmental concerns associated with agro-industrial waste disposal but also contributes to sustainable livestock production. This dual benefit is achieved through bioconversion processes such as microbial fermentation, which enhance the nutritional profile of the by-products while reducing waste and environmental burden. For example, fermentation of citric acid residues with *Bacillus subtilis* I9 has been reported to increase crude protein content by 21.89% and reduce crude fiber by 10.86%, improving feed quality for livestock [15]. Similarly, treatments with lactic acid bacteria and fibrolytic enzymes have further enhanced digestibility and protein content [16]. In practical applications, the inclusion of yeast waste-fermented citric residues in beef cattle diets has demonstrated improvements in protein intake, nutrient digestibility, and ruminal microbial activity [17].

In addition, studies in swine and poultry production have shown that citrus-based feed additives can reduce CO₂ emissions and water consumption, contributing to more environmentally sustainable animal agriculture [18]. These outcomes align with circular economy principles by transforming waste into high-value resources and minimizing dependence on conventional feedstuffs, thus mitigating food-feed competition [19, 20]. However, no studies have simultaneously investigated the combined effects of yeast waste fermentation, fiber-degrading enzyme supplementation, and pelleting on the utilization of citric acid residues in ruminant diets.

Therefore, the objective of this study was to evaluate the effects of pelleted citric waste fermented with yeast waste and supplemented with fiber-degrading enzymes, replacing 75% of soybean meal in concentrate, on gas production kinetics and in vitro digestibility.

## Results

### In vitro kinetics of gas production

According to Table 1, the interaction effects of citric waste fermented with yeast waste(CWYW) and different levels of fiber-degrading enzyme supplementation significantly influenced all gas production parameters (*P* < 0.01, *P* < 0.05).

**Table 1:** Effect of Fiber-degrading enzyme combined with citric waste fermented yeast waste supplementation on gas production kinetics

Treatment	Enzyme (%)	Gas kinetics				
		a	b	c	|a| + b	Total gas (ml)
Control	0	−7.35^cd^	95.24^a^	0.051^c^	101.99^a^	93.47^a^
CWYW-Powder	0	−7.93^d^	83.78^de^	0.058^b^	91.15^d^	79.47^e^
	0.2	−6.69^bcd^	86.63^d^	0.056^b^	93.33^cd^	83.27^d^
	0.4	−4.11^a^	80.51^e^	0.043^e^	85.34^e^	79.20^e^
	0.6	−4.61^a^	71.15^f^	0.049^cd^	76.72^f^	68.20^f^
CWYW-Pellet	0	−7.69^d^	82.24^e^	0.073^a^	90.35^d^	78.67^e^
	0.2	−6.77^bcd^	89.89^c^	0.050^c^	96.75^c^	85.87^c^
	0.4	−6.07^b^	93.49^ab^	0.049^cd^	99.56^ab^	92.20^ab^
	0.6	−6.20^bc^	91.66^bc^	0.048^d^	97.44^abc^	90.27^b^
SEM		0.32	1.09	0.001	1.56	0.81
Contrast						
Control VS Other		*P* < 0.05	*P* < 0.01	*P* < 0.05	*P* < 0.01	*P* < 0.01
Linear		*P* < 0.05	*P* < 0.01	*P* < 0.01	*P* < 0.01	*P* < 0.01
Quadratic		0.53	0.39	*P* < 0.01	*P* < 0.05	*P* < 0.05
Cubic		0.11	0.31	*P* < 0.01	*P* < 0.01	0.12

^a–f^ Means within a column bearing different superscripts differ (*P* < 0.05). a = gas produced from the immediately soluble fraction; b = gas produced from the insoluble but fermentable fraction; c = fractional rate constant of gas production from fraction b; |a| + b = estimated potential gas production (|a| + b); SEM = standard error of the mean (*n* = 4). CWYW = citric-waste-fermented yeast waste; the Control diet contained soybean meal as the sole protein source. Factor A = physical form of CWYW (powder vs. pellet); Factor B = inclusion level of fiber-degrading enzyme (0, 0.2, 0.4, or 0.6% of substrate DM). “Control vs Other” compares the soybean-meal control with all CWYW diets; linear, quadratic, and cubic rows are orthogonal polynomial contrasts across enzyme levels within each CWYW form

For gas production derived from water-soluble substances (a), significant differences were found between the control and CWYW groups. The control group exhibited a value of − 7.35, while the CWYW + enzyme treatments averaged − 6.26 (*P* < 0.05). The highest a values were observed in CWYW-powder supplemented with 0.4% and 0.6% enzyme (–4.11 and − 4.61, respectively), whereas the lowest values were recorded in CWYW-powder and CWYW-pellet without enzyme supplementation (–7.93 and − 7.69, respectively). A significant linear increase in a value was associated with increasing enzyme levels (*P* < 0.05).

For gas derived from water-insoluble substances (b), the control group recorded the highest value (95.26 mL), which was significantly greater than the average value of the CWYW + enzyme groups (84.92 mL) (*P* < 0.01). Among CWYW treatments, the highest b values were obtained from CWYW-pellet supplemented with 0.2%, 0.4%, and 0.6% enzyme (89.89, 93.49, and 91.66 mL, respectively), while the lowest b value was found in CWYW-powder with 0.6% enzyme (71.15 mL). A linear trend was observed across enzyme levels for parameter b (*P* < 0.01).

In terms of gas production rate (c), cumulative gas production (IaI + b), and total gas at 96 h, all values in the control group were significantly higher than those in CWYW + enzyme treatments. The control group exhibited c, IaI + b, and 96-h total gas values of 0.051 mL/h, 101.99 mL, and 93.47 mL, respectively, while the CWYW + enzyme groups had respective averages of 0.054 mL/h, 91.33 mL, and 82.14 mL (*P* < 0.01, *P* < 0.05).

The highest gas production rate (c) was observed in CWYW-pellet without enzyme (0.073 mL/h), whereas the lowest was recorded in CWYW-powder with 0.4% enzyme (0.043 mL/h). The highest cumulative gas production (IaI + b) occurred in CWYW-pellet with 0.0–0.6% enzyme and CWYW-powder with 0.0% and 0.2% enzyme (90.35, 96.75, 99.56, 90.27, 91.15, and 93.33 mL, respectively). The lowest value was found in CWYW-powder with 0.6% enzyme (76.72 mL). Both parameter c and IaI + b followed a significant cubic contrast pattern (*P* < 0.01). The highest total gas production at 96 h was recorded in CWYW-pellet supplemented with 0.4% and 0.6% enzyme (92.20 and 90.27 mL). In contrast, the lowest was found in CWYW-powder with 0.6% enzyme (68.20 mL), showing a quadratic contrast pattern (*P* < 0.05).

### Alterations in in vitro feed degradability and ruminal fermentation

According to Table 2, the in vitro dry matter digestibility (IVDMD) at 24 and 48 h, and the in vitro organic matter digestibility (IVOMD) at 24 h in the control group differed significantly from the treatments that included citric acid by-product fermented with yeast waste (CWYW) combined with fiber-degrading enzyme supplementation (*P* < 0.01, *P* < 0.05). However, the interaction between CWYW form and enzyme levels did not significantly affect either IVDMD or IVOMD.

**Table 2:** Effect of Fiber-degrading enzyme combined with citric waste fermented yeast waste supplementation on digestibility

Treatment	Enzyme (%)	IVDMD, (% DM)		IVOMD, (% DM)	
		24 h.	48 h.	24 h.	48 h.
Control	0	62.58	64.34	86.73	86.01
CWYW-Powder	0	55.89	55.62	80.54	84.30
	0.2	60.29	61.63	84.27	85.32
	0.4	60.65	63.43	86.55	86.00
	0.6	60.47	61.86	85.23	84.92
CWYW-Pellet	0	54.72	55.37	82.15	84.48
	0.2	57.33	60.02	85.34	85.90
	0.4	60.07	62.58	87.57	86.47
	0.6	59.79	61.52	86.44	85.08
SEM		0.71	0.44	0.78	0.81
Contrast					
Control VS Other		*P* < 0.01	*P* < 0.01	*P* < 0.05	0.42
Linear		0.41	0.28	0.81	0.98
Quadratic		0.42	0.60	0.75	0.76
Cubic		0.17	0.22	0.96	0.95
Single factor					
Factor A		*P* < 0.05	*P* < 0.01	*P* < 0.05	0.55
Factor B linear		*P* < 0.01	*P* < 0.01	*P* < 0.01	0.34
Factor B quadratic		*p* < 0.01	*p* < 0.01	*P* < 0.01	0.05
Factor B cubic		0.94	0.50	0.40	0.62

^a–f^Means within a column bearing different superscripts differ (*P* < 0.05). IVDMD = in-vitro dry-matter digestibility; IVOMD = in-vitro organic-matter digestibility; SEM = standard error of the mean (*n* = 4)

Factor definitions, contrasts, and treatment descriptions are as given for Table 1

Regarding the main effect of feed form, CWYW in powder form resulted in significantly higher IVDMD at both 24 and 48 h, and IVOMD at 24 h, compared to CWYW in pelleted form (*P* < 0.05, *P* < 0.01). In addition, enzyme supplementation level significantly influenced IVDMD (24 and 48 h) and IVOMD (24 h), showing a quadratic response pattern (*P* < 0.01), with the highest values observed at the 0.4% enzyme inclusion level. No significant differences in IVOMD were detected at 48 h across all treatments.

As shown in Table 3, the control group exhibited significantly higher ruminal pH at 48 h (6.74) and ammonia-nitrogen (NH₃-N) concentrations at 24 and 48 h (8.73 and 9.54 mg/dL, respectively) compared to treatments receiving CWYW combined with fiber-degrading enzymes, which recorded values of 6.71, 7.89, and 8.66 mg/dL, respectively (*P* < 0.05, *P* < 0.01).

**Table 3:** Effect of Fiber-degrading enzyme combined with citric waste fermented yeast waste supplementation on ruminal pH and ammonia nitrogen

Treatment	Enzyme (%)		Ruminal pH		Ammonia Nitrogen (mg/dL)	
			24 h.	48 h.	24 h.	48 h.
Control		0	6.75	6.74^a^	8.73	9.54^a^
CWYW-Powder		0	6.78	6.71^bc^	7.06	7.56^d^
		0.2	6.74	6.73^ab^	7.38	7.95^d^
		0.4	6.73	6.72^bc^	8.62	9.39^ab^
		0.6	6.76	6.75^a^	8.30	8.87^b^
CWYW- Pellet		0	6.74	6.73^ab^	7.21	8.50^c^
		0.2	6.76	6.71^bc^	7.55	8.62^c^
		0.4	6.75	6.70^c^	8.68	9.51^a^
		0.6	6.75	6.69^c^	8.31	8.89^b^
SEM			0.02	0.01	0.15	0.17
Contrast						
Control VS Other			0.89	*p* < 0.05	*p* < 0.01	*p* < 0.01
Linear			0.46	*P* < 0.01	0.61	*P* < 0.05
Quadratic			0.22	0.82	0.89	0.76
Cubic			0.86	*P* < 0.05	0.84	0.53
Single Factor						
Factor A			0.70	*P* < 0.05	0.37	*P* < 0.01
Factor B linear			0.63	0.36	*P* > 0.01	*P* < 0.01
Factor B quadratic			0.5	0.36	*P* < 0.01	*P* < 0.01
Factor B cubic			0.73	0.09	*P* < 0.01	*P* < 0.01

Factor A: form of CWYW (powder and pellet); Factor B: Level of enzyme % DM (0, 0.2, 0.4, and 0.6%). ^a–d^Means within a column bearing different superscripts differ (*P* < 0.05). Ammonia-N is expressed in mg dL⁻¹; SEM = standard error of the mean (*n* = 4). Factor A = CWYW form (powder vs. pellet); Factor B = enzyme level (0, 0.2, 0.4, 0.6% of substrate DM). Polynomial contrasts evaluate linear, quadratic, and cubic responses to increasing enzyme inclusion; “Control vs. Other” compares the soybean-meal control with all CWYW diets

The interaction between CWYW form and enzyme level significantly affected ruminal pH at 48 h and NH₃-N concentration at 48 h, although the differences were relatively small. A significant cubic trend was observed for both parameters (*P* < 0.05).

The highest NH₃-N concentrations at 48 h were observed in treatments with CWYW-powder supplemented with 0.4% and 0.6% enzyme, and CWYW-pellet supplemented with 0.4% and 0.6% enzyme (9.39, 8.87, 9.51, and 8.89 mg/dL, respectively). The lowest concentrations occurred in CWYW-powder with 0.0% and 0.2% enzyme supplementation (7.56 and 7.95 mg/dL, respectively). Furthermore, NH₃-N concentration at 24 h increased linearly with enzyme supplementation level (*P* < 0.05), and contrast analysis revealed a significant cubic response (*P* < 0.01).

### Volatile fatty acid profile

As shown in Table 4, the total volatile fatty acid (VFA) concentration at 48 h in the control group differed significantly from that in the CWYW + enzyme supplementation groups. Additionally, the VFA profile, including acetic acid (C2), propionic acid (C3), and butyric acid (C4) at 24 h, showed significant differences among treatments (*P* < 0.01). Treatments involving CWYW powder with 0% and 0.2% enzyme and CWYW pellet with 0.2% enzyme exhibited higher total VFA concentrations compared to CWYW powder and pellet supplemented with 0.4% and 0.6% enzyme, displaying a significant cubic trend (*P* < 0.01).

**Table 4:** Effect of Fiber-degrading enzyme combined with citric waste fermented yeast waste supplementation on volatile fatty acid

Treatment	Enzyme (%)	Total VFA (mM)		Acetic acid %		Propionic acid %		Butyric acid %	
		24 h.	48 h.	24 h.	48 h.	24 h.	48 h.	24 h.	48 h.
Control	0	105.32^b^	130.38	53.26^c^	58.26	30.43^a^	26.28	16.31^a^	15.46
CWYW – Powder	0	107.58^ab^	116.59	59.15^a^	59.64	26.59^cd^	25.82	14.25^cd^	14.54
	0.2	109.37^a^	120.63	58.47^a^	58.28	27.01^c^	26.79	15.29^b^	14.93
	0.4	99.06^c^	116.15	57.14^b^	58.82	27.34^c^	26.51	15.53^ab^	14.67
	0.6	100.26^c^	117.27	57.03^b^	59.46	29.06^b^	25.10	13.91^d^	15.44
CWYW – Pellet	0	103.60^bc^	121.49	59.59^a^	58.79	25.44^d^	25.89	14.97^bc^	15.31
	0.2	105.90^ab^	119.31	58.80^a^	58.82	26.27^cd^	25.15	14.93^bc^	16.04
	0.4	99.69^c^	118.81	58.77^a^	58.87	26.30^cd^	25.65	15.63^ab^	15.48
	0.6	100.48^c^	117.46	57.31^b^	58.06	26.80^cd^	26.02	15.12^bc^	15.92
SEM		1.43	1.97	0.46	0.64	0.46	0.61	0.27	0.31
Contrast									
Control VS Other		0.19	*P* < 0.01	*P* < 0.01	0.40	*P* < 0.01	0.53	*P* < 0.01	0.62
Linear		*P* < 0.01	0.27	*P* < 0.01	0.61	*P* < 0.01	0.71	0.83	0.09
Quadratic		0.61	0.72	0.99	0.53	0.46	0.49	*P* < 0.01	0.93
Cubic		*P* < 0.01	0.37	0.84	0.52	0.35	0.75	0.13	0.06
Single Factor									
Factor A		0.10	0.28	*P* < 0.05	0.38	*P* < 0.01	0.41	0.06	*P* < 0.01
Factor B linear		*P* < 0.01	0.27	*P* < 0.01	0.61	*P* < 0.01	0.71	0.83	0.09
Factor B quadratic		0.61	0.72	0.99	0.53	0.46	0.49	*P* < 0.01	0.93
Factor B cubic		*P* < 0.01	0.37	0.84	0.52	0.35	0.75	0.13	0.06

^a–d^Means within a column bearing different superscripts differ (*P* < 0.05). Total VFA = sum of acetate, propionate, butyrate, and minor acids (mM); individual acids are expressed both as % of total VFA and as absolute concentrations (mM); SEM = standard error of the mean (*n* = 4). Factor and contrast definitions are identical to those in Table 1

At 24 h, the highest C2 concentrations were recorded in CWYW powder supplemented with 0% and 0.2% enzyme and CWYW-pellet with 0%, 0.2%, and 0.4% enzyme (59.15%, 58.47%, 59.59%, 58.80%, and 58.77%, respectively). In contrast, the lowest C2 values were observed in CWYW-powder with 0.4% and 0.6% enzyme and CWYW-pellet with 0.6% enzyme (57.14%, 57.03%, and 57.31%, respectively), indicating a significant linear decrease (*P* < 0.01).

The highest C3 concentration (29.06%) was observed in the CWYW-pellet supplemented with 0.6% enzyme, and the increase was significant and linear with increasing enzyme levels (*P* < 0.01). Conversely, the lowest C4 concentration (13.91%) occurred in the CWYW-pellet with 0.6% enzyme, exhibiting a significant quadratic trend (*P* < 0.01).

No significant interaction effects between CWYW type and enzyme supplementation were observed on total VFA, C2, C3, or C4 concentrations at 48 h. However, the main effect of CWYW form (Factor A) significantly influenced C4 concentration (*P* < 0.01).

## Discussions

While the application of natural additives in animal feed encompasses a wide range of substances and mechanisms, this study focuses specifically on the synergistic potential of agro-industrial by-products (CWYW) and enzymatic treatment. The findings reported herein demonstrate that fiber-degrading enzyme inclusion significantly affected in vitro gas production kinetics and digestibility. Increasing the level of enzyme supplementation led to a higher gas production from the soluble fraction (a). This is attributed to enzymatic pre-hydrolysis, which facilitates the breakdown of complex feed particles, thereby releasing more soluble carbohydrates for immediate utilization. Concurrently, the gas produced from the insoluble fraction (b) decreased, particularly in treatments with CWYW powder combined with 0.4% and 0.6% enzyme levels, indicating improved feed degradation efficiency of the fibrous components. These findings are consistent with previous research [21], which reported increases in parameters a, c, and |a| + b, with no significant change in b. Interestingly, in this study, the rate of gas production (c) was inversely related to the level of enzyme supplementation, with the highest rate observed in diets without enzyme addition. This may be explained by the fact that enzymatic pre-treatment hydrolyzes the most accessible substrates before incubation, leaving the slower-degrading fractions for microbial fermentation, a mechanism supported by [22], who found a marked decrease in c as enzyme levels increased. The elevated values of |a| + b and cumulative gas production were likely due to enhanced starch and fiber degradation, providing substrates that supported microbial proliferation [12].

Regarding dry matter (IVDMD) and organic matter digestibility (IVOMD), the control group (soybean meal) exhibited superior digestibility compared to the CWYW-treated groups. This reduction may be due to replacing soybean meal with CWYW, which increases the proportion of indigestible fiber and thereby reduces nutrient digestibility [23]. Similarly, it was reported that the inclusion of more than 10% citric acid by-products decreased dry matter digestibility. Structural carbohydrate components in CWYW have also been shown to limit digestibility [12]. Additionally, CWYW in powdered form showed greater digestibility than the pelleted form, likely due to its finer texture, which increases surface area for microbial attachment and fermentation. In contrast, the dense pellet structure likely slowed microbial access and degradation [24]. Also observed a slight reduction in IVDMD in pelleted TMR diets for lambs. However, IVOMD at 24 h appeared to improve with pelleting, possibly due to modification of the fiber structure during heating. This observation is consistent with findings from [25], who noted improved digestibility of neutral detergent fiber (NDF) in pelleted compared to non-pelleted diets.

Supplementation with fiber-degrading enzymes, particularly at 0.4%, improved both IVDMD and IVOMD. This optimal dose suggests that enzymatic hydrolysis effectively enhanced the breakdown of fibrous materials, allowing ruminal microbes to digest the feed more efficiently. Nonetheless, increasing the enzyme level to 0.6% resulted in a decline in digestibility. This non-linear response may be attributed to over-processing or substrate saturation, leading to competition between free enzymes and rumen microbes for attachment sites and nutrients, thereby reducing overall combined activity. This observation aligns with [26, 27], who reported that over-supplementation of fibrolytic enzymes could reduce nutrient digestibility in dairy cattle.

Ruminal pH values ranged from 6.73 to 6.78 at 24 h and slightly decreased to 6.69 to 6.75 at 48 h, indicating a time-dependent effect of feed fermentation on pH. The use of CWYW and enzyme supplementation did not significantly affect ruminal pH, consistent with the findings of [12, 17, 28], who reported that enzyme and CWYW supplementation had minimal impact on ruminal pH in dairy cattle. Replacing soybean meal with CWYW significantly reduced NH₃-N concentrations, which may be attributed to the higher fiber content in CWYW impeding protein digestion, coupled with a slightly higher crude protein content in the control diet [29]. Similarly, it was noted that high crude protein diets increased ruminal NH₃-N due to microbial degradation of protein into ammonia. While some of this ammonia is utilized for microbial protein synthesis, excess accumulates in the rumen. Moreover, the enzyme supplementation enhanced fiber breakdown and indirectly elevated NH₃-N concentrations, consistent with the observations of [30], who reported that enzyme supplementation in high-forage dairy diets did not improve digestibility but increased ruminal NH₃-N, potentially indicating enhanced proteolytic activity.

The effect of CWYW and enzyme supplementation on VFA profiles showed a notable trend. Although total VFA changes were minimal, the decline in acetate (C2) and butyrate (C4) coincided with an increase in propionate (C3). This shift towards higher propionate production is a positive indicator of improved energetic efficiency in rumen fermentation. It aligns with the known mode of action of fibrolytic enzymes, which facilitate the release of fermentable carbohydrates [31]. Overall, the limited differences at 48 h suggest that the impact of treatments on fermentation end-products is most pronounced during the early stages of digestion.

## Conclusions

The present study aimed to improve the nutritional value of citric acid by-products through fermentation with yeast waste (CWYW) and supplementation with varying levels of fiber-degrading enzymes, targeting a 75% replacement of soybean meal in concentrate diets. Among the treatments, CWYW supplemented with 0.4% enzyme yielded gas production kinetics, digestibility, and ruminal ammonia-nitrogen concentrations that were most comparable to those of the control diet. The inclusion of CWYW with enzyme supplementation did not significantly affect ruminal pH, total volatile fatty acids, or individual VFA profiles.

To validate the practical efficacy of CWYW as a protein source in ruminant nutrition, further in vivo studies should be conducted under controlled experimental or farm-scale production conditions.

## Materials and methods

The Institutional Animal Care and Use Committee of Khon Kaen University approved all methods and procedures (record no. IACUC-KKU-25/68).

### Experimental design

A 2 × 4 + 1 factorial arrangement within a completely randomized design (CRD) was employed to investigate the effects of citric acid by-product fermented with yeast waste (CWYW) and fiber-degrading enzyme supplementation on replacing 75% of soybean meal in concentrate diets. Factor A consisted of two physical forms of CWYW (powder and pellet), while Factor B was the inclusion of a fiber-degrading enzyme (Agal Pro) at four levels: 0%, 0.2%, 0.4%, and 0.6%. An additional treatment served as the control (soybean meal-based concentrate). In total, nine treatments were arranged as follows:

1. Control (Soybean meal only).
2. CWYW-Powder + 0% enzyme.
3. CWYW-Powder + 0.2% enzyme.
4. CWYW-Powder + 0.4% enzyme.
5. CWYW-Powder + 0.6% enzyme.
6. CWYW-Pellet + 0% enzyme.
7. CWYW-Pellet + 0.2% enzyme.
8. CWYW-Pellet + 0.4% enzyme.
9. CWYW-Pellet + 0.6% enzyme.

### Experimental diets

The ingredient and nutrient composition of the experimental diets is presented in Table 5.. The diets were formulated to be isonitrogenous (18% CP) and isoenergetic, with CWYW replacing 75% of soybean meal in the concentrate portion.

**Table 5.** Chemical composition of feed and ingredients

Ingredient	Control	Feed 1	Feed 2	CW	YW	CWYW-powder	CWYW-pellet	Rice straw
Casava chips	25	25	25	-	-	-	-	-
soybean meal	20	5	5	-	-	-	-	-
Palm kernel	20	20	20	-	-	-	-	-
Rice brand	13.5	14	14	-	-	-	-	-
Corn	20	20	20	-	-	-	-	-
CWYW-pellet	-	-	15	-	-	-	-	-
CWYW-powder	-	15	-	-	-	-	-	-
Urea	0.5	0	0	-	-	-	-	-
Pre-mix	0.5	0.5	0.5	-	-	-	-	-
dicalcium phosphate	0.5	0.5	0.5	-	-	-	-	-
Chemical Composition%								
Dry matter %	95	95	92	92	18	83	95	97
Organic matter %DM	94.49	94.13	93.89	84.7	79.8	88.26	87.13	83.26
Crude Protein % DM	18.56	18.30	18.53	7.96	16.43	43.67	49.86	4.71
Ether extract % DM	3.97	3.55	3.82	1.27	0.34	0.57	1.89	1.21
NDF % DM	24.19	28.21	35.71	56.49	1.16	37.9	42.29	72.43
ADF %DM	17.56	15.62	20.32	44.38	0.43	30.84	27.96	51.27
ADL %DM	1.03	1.18	1.92	9.59	-	5.89	7.46	8.91
Ca %DM	1.12	1.37	1.68	1.32	4.95	1.52	1.59	-
P %DM	0.16	0.16	0.16	0.14	0.16	0.39	0.39	-

*YW* Yeast waste, *CW* Citric waste, *CWYW* Citric waste fermented yeast waste, *DM* Dry matter in the diet, *CP* Crude protein, *EE* Ether extract, *NDF* Neutral detergent fiber, *ADF* Acid detergent fiber, *ADL* Acid detergent lignin, *Ca* Calcium, *P* Phosphorus, Pre-mix including: Vitamin A = 10,000,000 IU, Vitamin E = 70,000 IU, Vitamin D = 1,600,000 IU, Fe = 50 g, Zn = 40 g, Mn = 40 g, Co = 0.1 g, Cu = 10 g, Se = 0.1 g, I: 0.5 g

### Preparation of experimental treatments

Yeast waste was obtained from Mitr Phol Biofuel Co., Ltd., Chaiyaphum, Thailand, and citric waste was provided by Sammor Farm Citric Acid Industry, Udon Thani, Thailand. Citric waste fermented yeast waste (CWYW) was prepared by mixing yeast waste (100 mL) with a solution composed of molasses (20 mL) and urea (50 g), which was adjusted to a final volume of 100 mL using distilled water. The mixture was aerated for 16 h and then anaerobically fermented with citric waste in a 1:1 ratio for 14 days. The mixture was then dried for 48 h to reduce moisture below 10%. For the pellet form, the dried CWYW mixture was processed using a triple-roller pellet mill (Model: 3-roller heavy-duty type, Samyod Motor Co., Ltd., Bangkok, Thailand). The material was pelleted through a 4 mm die at 60–65 °C. The resulting pellets were then oven-dried at 60 °C for an additional 48 h to ensure proper hardening and moisture reduction for storage stability.

### Preparation of substrates and inoculation for in vitro incubation

Experimental diets were formulated using a concentrate containing 18% crude protein and rice straw as the roughage source. Both components were dried at 60 °C for 48 h, ground to pass through a 1-mm screen, and mixed in a 40:60 ratio (concentrate: roughage). A 0.5 g sample of the mixed diet was weighed into a 50 mL serum bottle. The fiber-degrading enzyme Agal Pro (Kerry Group Ltd., Listowel, Ireland), containing 1000 U alpha-galactosidase/g and 5700 U endo-1,4 beta-glucanase/g, was supplemented at 0.2%, 0.4%, and 0.6% of the substrate. The bottles were incubated at 39 °C in preparation for inoculation.

The in vitro digestion system was established following the modified gas production technique described by [32]. Buffer and mineral solutions were prepared in advance, combined in suction flasks, and continuously flushed with carbon dioxide (CO₂) for approximately one hour to establish anaerobic conditions. The resazurin color change from blue to light pink confirmed the absence of oxygen, and the buffered solution was maintained at 39 °C on a heated magnetic stirrer.

Ruminal fluid was collected from fistulated cattle by oral intubation using a vacuum pump (~ 1 L) and immediately transferred into a pre-warmed thermos flask for temperature maintenance during transport. In the laboratory, the ruminal fluid was filtered through four layers of cheesecloth into a 1000 mL measuring cylinder while being continuously flushed with CO₂. The filtered fluid was then mixed with the pre-warmed, CO₂-flushed buffer solution in a suction flask to prepare the inoculum.

Each serum bottle was flushed with CO₂ by inserting a needle through the rubber stopper to displace residual oxygen. Then, 40 mL of the ruminal-buffer inoculum was injected into each bottle using a 50 mL plastic syringe. After inoculation, the bottles were promptly returned to the incubator at 39 °C for gas production monitoring at predetermined time intervals.

## Data collection

### Gas volume measurement

The volume of gas produced during incubation was measured using a horizontal glass syringe connected to a rubber tube and needle. The needle was inserted through the rubber stopper of each incubation bottle, allowing the accumulated gas to push the syringe plunger until it stabilized. Once gas displacement ceased, the gas volume was recorded at specific time intervals as defined in the experimental protocol. The recorded values were used for subsequent calculations and analysis.

### Chemical composition and fiber analysis

The concentrate and roughage feeds were analyzed for chemical composition using standard procedures [33], including dry matter (DM), ash, and crude protein (CP). In addition, fiber fractions, namely, neutral detergent fiber (NDF) and acid detergent fiber (ADF), were analyzed as described in [34]. Gas production was recorded at multiple time points: 0, 1, 2, 3, 4, 6, 8, 10, 12, 24, 48, 72, and 96 h. The resulting data were used to model gas production kinetics using the equation proposed by [35] :

$$Y\;=\;a\;+\;b\;(1\;-\;e^{\left(-ct\right)})$$

Where:

- *Y* = gas production at time *t*.
- *a* = gas production from the soluble fraction.
- *b* = gas production from the insoluble but fermentable fraction.
- *c* = gas production rate constant.
- *t* = incubation time.
- *(a + b)* = potential gas production capacity.

### In vitro digestibility (IVDMD and IVOMD)

At 12 and 24 h of incubation, one bottle per treatment was randomly selected and immediately frozen at − 20 °C to halt microbial activity until analysis. Upon thawing, the undigested residue was collected and analyzed for dry matter using the [33] method. The organic matter (OM) content was determined by ashing the samples at 500 °C for 3 h. The dry matter digestibility (IVDMD) and organic matter digestibility (IVOMD) were calculated using the following formulas:

$$IVDMD\;(\%)\;=\frac{\lbrack(initial\;dry\;sample\;x\;\%DM\;of\;sample)\;-\;(DM\;residue\;-\;Blank)\rbrack\;}{\lbrack(initial\;dry\;sample\;x\;\%\;DM)\rbrack}\times100$$

$$IVOMD\;(\%)\;\;=\frac{\lbrack(DM\;weight\;x\;\%\;OM\;of\;sample)\;-\;(OM\;residue\;-\;Blank)\rbrack}{\lbrack(DM\;weight\;x\;\%\;OM\;of\;sample)\rbrack}\times100$$

### Determination of Ammonia-Nitrogen and ruminal pH

Ammonia-nitrogen (NH₃-N) concentrations were determined using the colorimetric method of [36]. A reaction mixture consisting of 40 µL of ruminal fluid, 2,500 µL of phenol color reagent, and 2,000 µL of alkaline hypochlorite was prepared in a 15-mL test tube and mixed using a vortex mixer. The mixture was then incubated in a water bath at 37 °C for 10 min. A blue-green color developed over time, and absorbance was measured at 630 nm using a UV/Vis spectrophotometer (London, UK). NH₃-N concentrations were calculated from a standard curve with concentrations of 0, 1, 2, 4, 8, and 16 mg/dL and reported in mg/dL.

### Volatile fatty acid (VFA) analysis

Frozen ruminal fluid samples were thawed and centrifuged at 16,000 rpm for 10 min. VFA concentrations following the technique outlined by [37]. After centrifugation, 0.3 mL of 50% sulfuric acid, 0.8 mL of 2–5-methylvaleric acid, and 1.6 mL of diethyl ether were added to the supernatant. The mixture was centrifuged at 2,500 rpm for 15 min to separate phases. A 1-mL aliquot of the ether extract was transferred to a pre-weighed microtube and allowed to stand for 5 min. The extract was then injected into a gas chromatograph (model HP6890, Hewlett Packard) equipped with a 10-µL injection port. One µL of sample was injected at an injector temperature of 200 °C and a box heater temperature of 150 °C. The column used was a Restek Stabilwax (30 m × 250 μm × 0.25 μm), with the oven maintained at 120 °C. Detector settings included a temperature of 200 °C, airflow at 400 mL/min, H₂ at 40 mL/min, N₂ at 25 mL/min, and He carrier gas at 1.5 mL/min.

### Statistical analysis

Data were analyzed using analysis of variance (ANOVA) following a 2 × 4 + 1 factorial arrangement in a completely randomized design (CRD), employing the General Linear Model (GLM) procedure of SAS software (SAS Institute Inc., 2010). The model used was:

$$Yij\;=\mu+\tau i+\varepsilon ij$$ 

Where Yij is the observed value, µ is the overall mean, τ_i_ is the fixed effect of treatment (CWYW form and enzyme level), and ɛij is the random error term. Data are presented as means ± standard error of the mean (SEM). Treatment means were compared using Duncan’s New Multiple Range Test (DMRT) [38]. Orthogonal polynomial contrasts were used to evaluate the treatment response patterns linear, quadratic, and cubic at a 95% confidence level (*P* < 0.05).

## Data Availability

Not applicable (this manuscript does not report data generation or analysis).

## Abbreviations

ADF: Acid detergent fiber
ADL: Acid detergent lignin
C2: Acetic acid
C3: Propionic acid
C4: Butyric acid
CF: Crude fiber
CP: Crude protein
CW: Citric waste
CWYW: Citric waste fermented with yeast waste
DM: Dry matter
EE: Ether extract
IVDMD: In vitro dry matter digestibility
IVOMD: In vitro organic matter digestibility
NDF: Neutral detergent fiber
NH₃-N: Ammonia nitrogen
OM: Organic matter
VFA: Volatile fatty acid
YW: Yeast waste
